# Circadian Rhythm-Dependent Alterations of Gene Expression in *Drosophila* Brain Lacking Fragile X Mental Retardation Protein

**DOI:** 10.1371/journal.pone.0037937

**Published:** 2012-05-24

**Authors:** Shunliang Xu, Mickael Poidevin, Enji Han, Jianzhong Bi, Peng Jin

**Affiliations:** 1 Department of Neurology, 2nd Hospital of Shandong University, Jinan City, Shandong Province, People's Republic of China; 2 Department of Neurology, Qilu Hospital of Shandong University, Jinan City, Shandong Province, People's Republic of China; 3 Department of Human Genetics, Emory University School of Medicine, Atlanta, Georgia, United States of America; Imperial College London, United Kingdom

## Abstract

Fragile X syndrome is caused by the loss of the *FMR1* gene product, fragile X mental retardation protein (FMRP). The loss of FMRP leads to altered circadian rhythm behaviors in both mouse and *Drosophila*; however, the molecular mechanism behind this phenomenon remains elusive. Here we performed a series of gene expression analyses, including of both mRNAs and microRNAs (miRNAs), and identified a number of mRNAs and miRNAs (miRNA-1 and miRNA-281) with circadian rhythm-dependent altered expression in *dfmr1* mutant flies. Identification of these RNAs lays the foundation for future investigations of the molecular pathway(s) underlying the altered circadian rhythms associated with loss of dFmr1.

## Introduction

Fragile X syndrome (FXS), one of the most common forms of inherited mental retardation, is characterized by mental retardation of variable severity, autistic behavior, macroorchidism in adult males, characteristic facial features, and hyperextensible joints [1]. FXS is mainly caused by a massive CGG trinucleotide repeat expansion (usually more than 200 repeats) within the 5′ untranslated region (UTR) of the fragile X mental retardation 1 gene (*FMR1*), which results in abnormal DNA methylation of both a nearby CpG island and the repeat itself; as a result, the transcription of *FMR1* is silenced [2]–[6]. Identification of other mutations (e.g., deletions in patients with the typical phenotype) has confirmed that *FMR1* is the only gene involved in the pathogenesis of fragile X syndrome, and the loss of the *FMR1* product, fragile X mental retardation protein (FMRP), causes fragile X syndrome [7]–[9].

In mammals, FMRP, along with its autosomal paralogs, the fragile X-related proteins FXR1P and FXR2P, constitute a well-conserved, small family of RNA-binding proteins (the fragile X-related gene family) that share over 60% amino acid identity and contain two types of RNA-binding motifs: two ribonucleoprotein K-homology domains (KH domains) and a cluster of arginine and glycine residues (RGG box) [10], [11]. Unlike their mammalian counterparts, the fly genome harbors a single *Fmr1* gene homolog, also referred to as *dFmr1*. Sequence comparisons show a high level of similarity between the functional domains of fly and mammalian *Fmrp*, with an overall 56% similarity and 35% identity [12], [13]. FMRP is found to form a messenger ribonucleoprotein (mRNP) complex that associates with translating polyribosomes [14]. FMRP is proposed to be involved in synaptic plasticity via the regulation of mRNA transportation and translation [15]. In addition, FMRP is associated with *Argonaute 2* (*AGO2*) and the RNA-induced silencing complex (RISC) [16]–[18]. Work from several groups suggests that FMRP can regulate the translation of specific mRNAs via the microRNA pathway [18]–[20].

To understand the molecular pathogenesis of fragile X syndrome, researchers have generated and extensively studied both mouse and *Drosophila* models. Besides the deficits in learning and memory in these models, one consistent behavioral abnormality they share is altered circadian rhythm behaviors, which potentially mimics the sleep abnormalities seen in patients with fragile X syndrome [21]–[24]. Sleep disorders are common in FXS patients, with problems including shorter sleep duration, greater variation in sleep duration, longer night waking episodes, and sleep timing problems [25]. These disturbances are believed to correlate with circadian rhythm dysregulation.

Circadian rhythm describes the approximately 24-hour cycles generated by a master pacemaker located in the suprachiasmatic nuclei (SCN) of the anterior hypothalamus of the mammalians and in the ventral lateral neurons (LNvs) of *Drosophila* [26]. Rhythms are manifest in such processes as locomotor activity and feeding behavior, sleep/wake patterns, and a variety of physiological and metabolic pathways. These circadian outputs are regulated by a central pacemaker, which receives environmental inputs and keeps circadian time. Although altered circadian rhythms have been seen in *Drosophila* model of FXS, however, there was no defect in the expression of clock components and the underlying molecular mechanism remains elusive [22]. Here we performed a series of gene expression analyses, including of both mRNAs and microRNAs (miRNAs), and identified a number of mRNAs and miRNAs (miRNA-1 and miRNA-281) with circadian rhythm-dependent altered expression in *dfmr1* mutant flies. These observations support a role for dFMR1 in the miRNA pathway and implicate the altered expression of selective miRNAs in the circadian abnormalities associated with the loss of dFMR1 in fly.

## Materials and Methods

### Drosophila strain treatment and RNA sample preparation

*Drosophila dfmr1* null mutant strains, dFmr1-Del3-21/TM6C, Kr:GFP, were described previously. Flies were raised with standard food at 25°C. Male flies were selected and entrained to a 12 h∶12 h Light-Dark (LD) cycle in a 25°C incubator for 4 days, and free run on the fifth day [consent dark (DD)]. Circadian Time 00 (CT00) refers to the subjective point when lights come on. The flies were harvested every 4 hours at time points CT00, CT04, CT08, CT12, CT16, CT20, and CT24 on the fifth day, transferred to Eppendorf tubes, and frozen in dry ice immediately. The fly heads were collected and homogenized in 1 ml Trizol reagent (Invitrogen).

### Microarray analyses

cRNA amplification and fluorescence labeling was performed according to the supplier's instructions using the Affymetrix 3′IVT kit (Affymetrix Technologies). The labeled target was combined and allowed to hybridize to probes on the *Drosophila* genome 2.0 array according to instructions. The arrays were washed using the Midi _euk2v3 fluidics protocol and scanned using the Microarray Scanner laser-based detection system. All normalizations were performed using default settings.

Image data were quantified using the Affymetrix expression console. All analysis was performed using Bayesian infinite mixture models as implemented in the *BBR* software, version 3.8.1 (http://linus.nci.nih.gov/BRB-ArrayTools.html), an integrated package for the visualization and statistical analysis of DNA microarray gene expression data. Gene expression data were normalized using the robust multi-array average (RMA) statistical algorithms built in BRB. All filtering parameters were turned off. Class comparison and cluster analysis were performed using the Bayesian infinite mixture models as implemented in the *BBR* software. Heat map was generated by clustering genes and arrays with complete linkage uncentered correlation using Cluster 3 and Java TreeView [27].

Significantly differentially expressed genes were annotated with functional assignments to help determine which gene categories were enriched with differentially expressed genes. Genes were annotated and biological processes analyzed using the Database for Annotation, Visualization and Integrated Discovery (DAVID) (http://david.abcc.ncifcrf.gov/) [28].

### Real-Time RT-PCR

TaqMan MicroRNA Assays detecting 72 known individual *Drosophila* miRNAs were obtained from ABI (ABI). cDNA was prepared with High-Capacity cDNA Reverse Transcription Kits (ABI; Cat#437496). The 15-µl reverse transcription reactions consisted of 10 ng of total RNA, 5 U MultiScribe Reverse Transcriptase, 0.5 mM of each dNTP, 1× reverse transcription buffer, 4 U RNase inhibitor, and nuclease-free water. This was performed at 16°C for 30 min and at 42°C for 30 min, terminated at 85°C for 5 min, and stored at 4°C until use in TaqMan assays. For real-time PCR of TaqMan MicroRNA Assays, we used 0.5 ul 20×TaqMan MicroRNA Assay Primer, 1.33 ul undiluted cDNA, 5 ul 2×TaqMan Universal PCR Master Mix, and 3.17 ul nuclease-free water. Each PCR reaction was performed in triplicate with MicroAmp optical 96-well plates using a 7500 Fast Real-Time PCR System (ABI), with reactions incubated at 95°C for 10 min, followed by 40 cycles of 95°C for 15 s, and 60°C for 1 min. Fluorescence readings were taken during the 60°C step. RQs were calculated using the ΔΔCt method, with 2S RNA TaqMan miRNA control assay as the endogenous control, and calibrated to the control samples.

Real-time PCR was conducted with 2× SYBR Green Master Mix (Applied Biosystems) as amplification of Period, Timeless, microRNA Precursor, and Primary Precursor. To prepare for the cDNA with SuperScript III First-Strand Synthesis System (Invitrogen) according to the manufacturer's instructions, Pri-miR1-Forward primer and Pre-miR1-Reverse Primer, Pri-miR281-Forward primer and Pre-miR281-Reverse Primer were used as specific primers for Period and Timeless using oligo(dT). Real-time PCR was performed with the following PCR parameters: 50°C for 2 min, 95°C for 10 min, then 95°C for 15 s, 60°C for 1 min for 40 cycles.

Drosophila-Period-Forward Primer 5′-GGGATCATATCGCACGTGGAC-3′

Drosophila-Period-Reverse Primer 5′-CTGCGGCCAATCAGGTCCTG-3′

Drosophila-Timeless-Forward Primer 5′-GCCTGGGCAATGAGCCATTC-3′

Drosophila-Timeless-Reverse Primer 5′-GAGGTGGAGGCTCTGACTGG-3′

Pre-miR1-Forward Primer 5′-GAGAGTTCCATGCTTCCTTGC-3′

Pre-miR1-Reverse Primer 5′-CCAGATTTCGCTCCATACTTC-3′

Pri-miR1-Forward Primer 5′-CTGTCCAAGTGAGTAGTGCCAC-3′

Pri-miR1-Reverse Primer 5′-AAGGCTGAATATGGGATGGTC-3′

Pre-miR281-Forward Primer 5′-CGAATAAGTGAATAAAGAGAGC-3′

Pre-miR281-Reverse Primer 5′-GAGCAATTCCATGACAGTGAT-3′

Pri-miR281-Forward Primer 5′-GTATGTATCTGTGCTGTCCAC-3′

Pri-miR281-Reverse Primer 5′-CACTTATTCGCATTTGGATTCG-3′

## Results

### The loss of Fmrp does not alter the expression of circadian rhythm master genes

**Figure 1:**
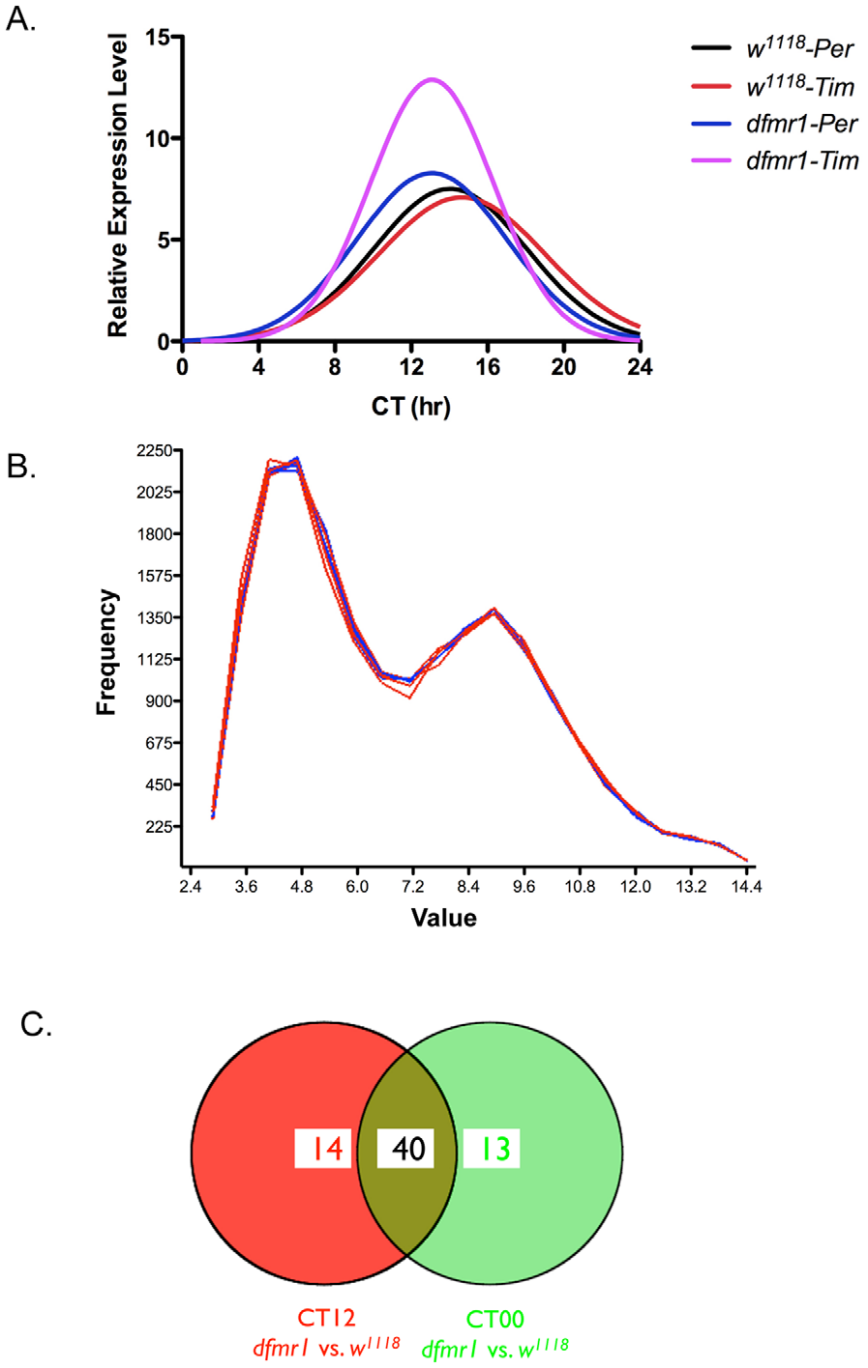
Gene expression profiling in wild-type and dfmr1 null mutant fly heads. **A.** Quantitative RT-PCR was used to measure the levels of the indicated transcripts in both *w^1118^* and *dfmr1* null fly heads between CT00 and CT24. **B.** Frequency distribution of gene expression levels in different genotypes. **C.** Venn diagram showing the number of genes with significant and consistent changes (≥1.5 fold) in *dfmr1* null mutant fly heads at CT00 and CT12, and the overlap between two time points.

Circadian clocks are controlled by transcriptional feedback loops in which several clock genes regulate their own and other clock genes' transcription [26]. In *Drosophila*, the first two clock genes identified were *period* and *timeless*, which are the core molecular components of the circadian clock [26]. Their transcriptional feedback loops contribute to the oscillation of master circadian rhythm. Given the circadian abnormalities associated with the loss of dFmr1, we entrained the male 2-day-old flies to a 12 h∶12 h Light/Dark (LD) cycle for 4 days and free run on the fifth day, and fly heads were collected every 4 hours on the fifth day. RNA was isolated from each time point and used for real-time RT-PCR to determine the expression of Period (Per) and Timeless (Tim). Similar expression patterns were observed between *w^1118^* and *dfmr1* null mutant flies, although the expression level of Tim at CT12 was significantly elevated in *dfmr1* mutant (Figure 1A). For the following expression profiling studies, we focused on 2 time points, CT00 and CT12.

### The loss of dFmr1 leads to circadian rhythm-dependent alteration of specific gene expression in Drosophila heads

**Figure 2:**
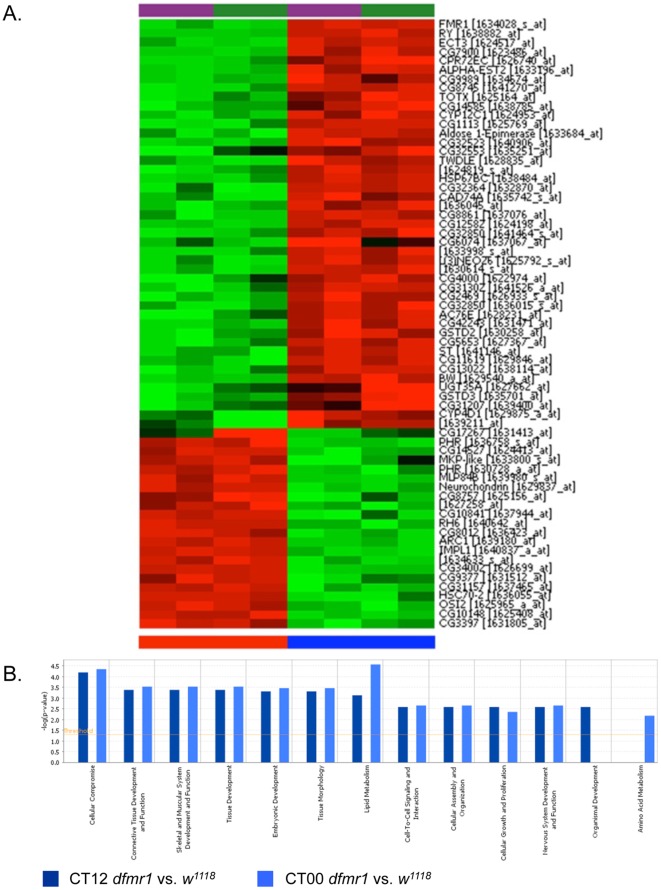
Altered gene expression in *dfmr1* mutant fly heads. **A.** Heat map of mRNAs with significant and consistent changes (≥1.5 fold) in *dfmr1* null mutant fly heads. Transcripts that were present at higher levels in each genotype are shown in progressively brighter shades of red, and ones that were expressed at lower levels are shown in progressively brighter shades of green. Bottom: Red represents *dfmr1* mutant, while blue indicates the wild-type (*w^1118^*). CT00-purple and CT12-Green. **B.** Enrichment of pathways associated with the altered gene expression at CT00 and CT12 in *dfmr1* null mutant fly heads.

To determine how the loss of dFmr1 alters gene expression in a circadian rhythm-dependent manner, we prepared the RNA samples in triplicate from both *w^1118^* and *dfmr1* mutant fly heads and analyzed the expression profiles using GeneChip® *Drosophila* Genome 2.0 arrays. Consistent with the previous findings, most of the genes are unchanged at both CT00 and CT12 (Figure 1B). At CT00, 53 genes (over 1.5-fold difference, p<0.001) display significant differences between *w^1118^* and *dfmr1* mutant, whereas at CT12, 54 genes (over 1.5-fold difference, p<0.001) were found with altered expression (Figure 1C and Figure 2A). Among them, 40 genes displayed consistent changes at both time points. Gene ontology analyses revealed distinct pathways that could be altered in the absence of *dFmr1* gene (Figure 2B).

**Figure 3:**
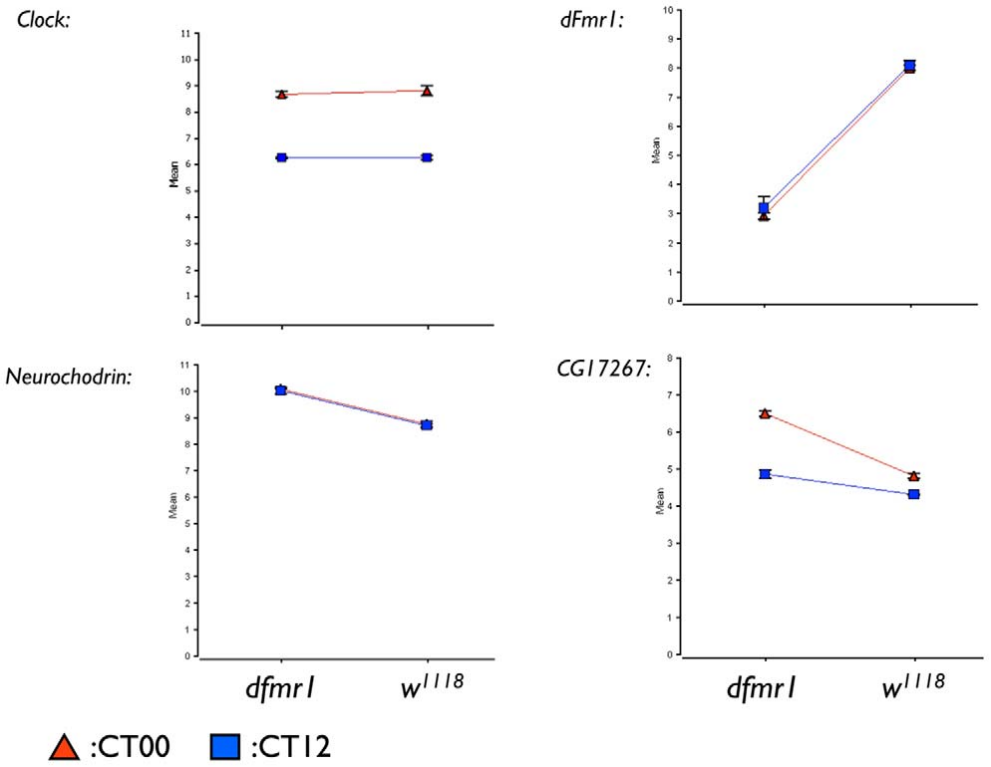
Validation of altered gene expression by real-time PCR. Quantitative RT-PCR was used to measure the levels of the indicated transcripts in both *w^1118^* and *dfmr1* null fly heads.

We further validated our findings using real-time RT-PCR. As shown in Figure 3, dFmr1 expression was abolished in *dfmr1* mutant flies, while Clock gene expression is not altered in *dfmr1* mutant flies. Neurochodrin was found to be consistently upregulated in the absence of dFmr1. Intriguingly, CG17267 was increased significantly only at CT00, which reflects a circadian rhythm-dependent alteration. Currently, there is no known biological function of CG17267, and it would be interesting to examine its potential involvement in circadian rhythms regulated by dFmr1.

### The loss of dFmr1 leads to altered expression and biogenesis of miR-1 and miR-281

**Figure 4:**
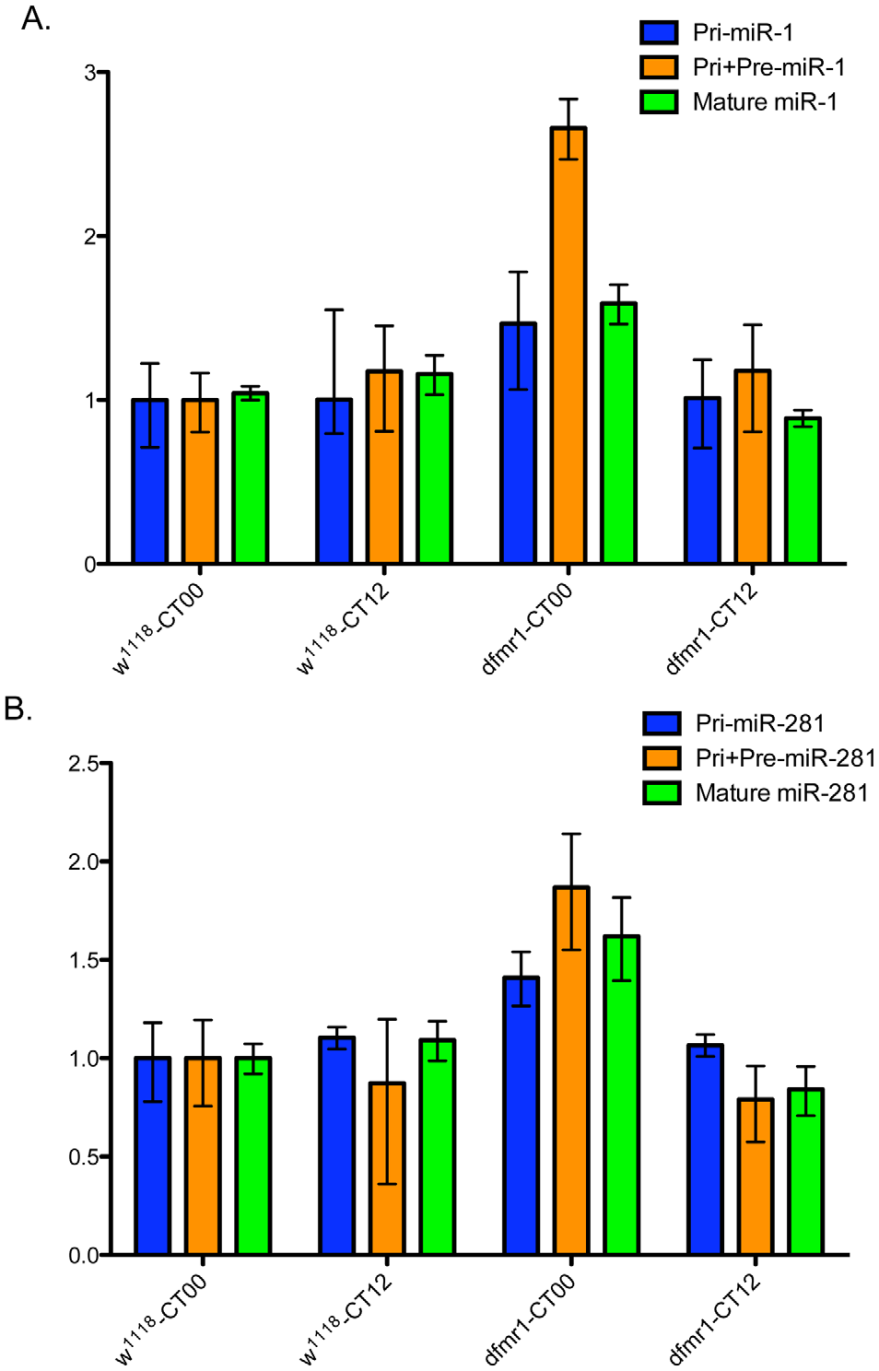
The loss of dFmr1 leads to altered expression and biogenesis of miR-1 and miR-281. Quantitative RT-PCR was used to measure the levels of pri-, pre-, and mature forms of miR-1 (A) and miR-281 (B) in *w^1118^* and *dfmr1* null fly heads. The relative expression levels as determined by ΔΔCt analyses are shown. Values are mean ± SD for triplicate samples. *: P<0.001.

Given that dFMR1 physically associates with AGO1, a key component in the miRNA pathway, we tried to determine in *Drosophila* whether any miRNAs could be involved in circadian regulation mediated by dFmr1 [18], [29]. To test this, TaqMan assays detecting 72 known individual miRNAs were used to analyze the expression of these microRNAs at CT00 and CT12 in both *w^1118^* and *dfmr1* mutant fly heads. Among these miRNAs, we saw significant changes of mature miR-1 and miR-281, in particular at CT00 (Figure 4A and 4B). Interestingly, while the ratio between CT00 and CT12 in *w^1118^* is unchanged, both miR-1 and miR-281 are significantly elevated at CT00, which displayed circadian rhythm-dependent expression in *dfmr1* mutant fly.

Given that dFMR1 is also found to be associated with Dicer, we further examined the processing of both miR-1 and miR-281 by real-time RT-PCR. We found that the precursor forms, but not the pri-form, of both miR-1 and miR-281 increased at CT00, indicating that the processing of these two miRNAs is altered in a circadian rhythm-dependent manner in the absence of dFmr1.

## Discussion

Fragile X syndrome (FXS) is characterized by mental retardation of variable severity, autistic behavior, macroorchidism in adult males, characteristic facial features, and hyperextensible joints [1]. FXS is caused by the loss of the *FMR1* gene product, fragile X mental retardation protein (FMRP) [7]–[9]. To understand the molecular pathogenesis of fragile X syndrome, researchers have generated and extensively studied both mouse and *Drosophila* models. Besides the deficits in learning and memory in these models, one consistent behavioral abnormality they share is altered circadian rhythm behaviors; however, the molecular mechanism behind this phenomenon remains elusive. Here we performed a series of gene expression analyses, including of both mRNAs and microRNAs (miRNAs), and identified a number of mRNAs and two miRNAs (miRNA-1 and miRNA-281) with circadian rhythm-dependent altered expression in *dfmr1* mutant flies.

Consistent with the notion that FMRP is a translational regulator, we did not detect any change of gene expression in a large number of genes at the mRNA level [30]. Interestingly, a third of the mRNAs that are altered either at CT00 or CT12 displayed the change only at one time point, which reflects a circadian rhythm-dependent alteration. How these changes could contribute to the altered circadian rhythms through the loss of dFmr1 warrants further investigation. It is also possible that the observed changes could be a direct consequence of a defect in regular sleep pattern rather than a direct consequence of the molecular clock, which would need further investigation. It would also be interesting to test whether dFMR1 associates with these mRNAs in a circadian rhythm-dependent manner, which has not been explored before. Finally, the biological functions of most mRNAs we identified are still unknown, and it might prove fruitful to examine their roles in circadian rhythms in general, as well.

Among the genes that we identified here, Neurochondrin has been shown involved in the regulation of MCHR1 signaling, and play a role in modulating melanin-concentrating hormone-mediated functions in vivo, including neuroendocrine, behaviors and circadian output [31], [32]. Neurochondrin could also interact with a subset of group I mGluRs, which has been implicated in fragile X syndrome [33], [34]. It would be interesting to determine whether Fmrp could directly bind to Neurochondrin mRNA and regulate its expression.

In this study, we also examine the profiles of all the known microRNAs in the context of circadian rhythms. In particular, the loss of dFmr1 led to the circadian rhythm-dependent alteration of miR-1 and miR-281 expression. This finding is particularly intriguing, since most of the previously published works on dFmr1 did not use entrained flies [35]. Our results indicate that dFmr1 could play a role in modulating expression and biogenesis in circadian rhythms. Since dFmr1 expression remains constant throughout the circadian cycle, it would be interesting to identify the protein(s) that could dynamically interact with dFMR1 and be involved in the modulation of the miRNA pathway. More importantly, we also need further investigation into whether there are such alterations in terms of miRNA processing in mammals.

In summary, we have performed systematic profiling of both mRNA and miRNA in both wild-type and *dfmr1* mutant fly heads and identified a subset of mRNAs and miRNAs that display circadian rhythm-dependent altered expression in *dfmr1* mutant flies, which will provide the foundation for future investigations into the molecular pathway(s) underlying the altered circadian rhythms caused by the loss of dFmr1.
